# Effect of assimilating CO_2_ observations in the Korean Peninsula on the inverse modeling to estimate surface CO_2_ flux over Asia

**DOI:** 10.1371/journal.pone.0263925

**Published:** 2022-02-18

**Authors:** Minkwang Cho, Hyun Mee Kim

**Affiliations:** Department of Atmospheric Sciences, Atmospheric Predictability and Data Assimilation Laboratory, Yonsei University, Seodaemun-gu, Seoul, Republic of Korea; George Mason University, UNITED STATES

## Abstract

To investigate the impact of two CO_2_ observation datasets obtained from the Korean Peninsula on the surface CO_2_ flux estimation over Asia, the two datasets are assimilated into the CarbonTracker (CT) inverse modeling system and the estimated surface CO_2_ fluxes are analyzed. Anmyeon-do (AMY) and Gosan (GSN) sites in the Korean Peninsula have observed surface CO_2_ mole fraction since the late 1990s. To investigate the effect of assimilating the additional Korean observations on the surface CO_2_ flux estimation over Asia, two experiments are conducted. The reference experiment (CNTL) only assimilates observations provided by National Oceanic and Atmospheric Administration (NOAA), while the other experiment (EXP1) assimilates both NOAA observations and two Korean observation datasets. The results are analyzed for 9 years from 2003 to 2011 in Asia region because both AMY and GSN datasets exist almost completely for this period. The annual average of estimated biosphere CO_2_ flux of EXP1 shows more flux absorption in summer and less flux emission from fall to spring compared to CNTL, mainly on Eurasia Temperate and Eurasia Boreal regions. When comparing model results to independent CO_2_ concentration data from surface stations and aircraft, the root mean square error is smaller for EXP1 than CNTL. The EXP1 yields more reduction on uncertainty of estimated biosphere CO_2_ flux over Asia, and the observation impact of AMY, GSN sites on flux estimation is approximately 11%, which is greater than other observation sites around the world. Therefore, the two CO_2_ observation sets in the Korean Peninsula are useful in reducing uncertainties for regional as well as global scale CO_2_ flux estimation.

## 1. Introduction

The annual mean surface air temperature of the earth has increased since the second industrial revolution in the late 19^th^ century. Global mean surface temperature (GMST) of the last decade (2011–2020) was approximately 1.09°C higher than that of the preindustrial period (1850–1900) [1]. The Paris Agreement adopted in 2015 aims at limiting the GMST rise below 2°C, or 1.5°C if possible, compared to the pre-industrial levels [2]. However, Intergovernmental Panel on Climate Change (IPCC) special report has stated that the temperature increase could surpass 1.5°C at the middle of this century without additional emission decrease, even if the current nationally determined contributions (NDCs) are achieved [3]. Therefore, it is important to estimate the precise sources and sinks of greenhouse gases in order to support the emission policies based on the scientific information as well as to manage the risks from the climate change.

CO_2_ is the most abundant component among the greenhouse gases in the troposphere. Since [4] attempted to estimate surface CO_2_ flux by assimilating observed CO_2_ mole fraction data in a model, many researchers have studied surface CO_2_ flux optimization using data assimilation (DA) approach. Surface CO_2_ flux estimation is an inverse modeling problem, known as an under-deterministic problem, which finds the solutions from relatively small number of observations. Thus, it needs prior surface CO_2_ flux information and more importantly observation data accumulated in long time period, so as to estimate the surface CO_2_ fluxes more precisely. Observations from the surface (i.e., flask observation and in situ observation) are commonly used in the inverse modeling. The spatial density of the surface CO_2_ observations is relatively high in North America and Europe, giving more reliable surface CO_2_ flux information of the two continents [5]. On the other hand, Asia, Africa, and South America have relatively low observation densities to well enough constrain the surface CO_2_ fluxes. To cover the deficiency of observation density in Asia, inverse modeling researches have been conducted by assimilating tower observations in Siberian region and aircraft observations over the globe into inverse models [6–9]. Satellite column CO_2_ (XCO_2_) data have also been used to supplement the spatial coverage of surface CO_2_ observations. The Greenhouse Gases Observing Satellite (GOSAT; [10, 11]) XCO_2_ data have been used in various inverse modeling studies [12–21]. The Orbiting Carbon Observatory-2 satellite (OCO-2; [22, 23]) XCO_2_ data also have been used in several studies [19, 15, 24–26]. Despite broad spatial coverage of satellite XCO_2_ data, the surface CO_2_ flux estimation using satellite XCO_2_ data shows large uncertainty depending on observation coverage, number of data, and retrieval algorithm [15, 17, 19]. Those studies have shown some improvements in optimizing surface CO_2_ flux in Asia, but more observation data are required to more precisely estimate the surface CO_2_ fluxes in terms of their spatial and temporal patterns as well as total annual budgets in different ecoregions over Asia.

Meanwhile, each country involved in the Paris agreement is obliged to set up its own contribution to greenhouse gas emission reduction and report its annual emission inventory to United Nations Framework Convention on Climate Change (UNFCCC) to check how much reduction is achieved. World Meteorological Organization (WMO) has established Integrated Global Greenhouse Gas Information System (IG3IS) platform, which combines traditional inventory reporting and inverse modeling results to support countries to make the inventory report with less uncertainties. [27] applied CO_2_ mole fraction data observed from two surface in situ observatories and shipboard across New Zealand to an inverse model, and revealed that the modeled CO_2_ flux absorption from indigenous forests in New Zealand is stronger than the absorption calculated from the inventory report. In the UK, CO_2_ observation from the tower network were assimilated into an inverse model and the estimated CO_2_ flux from the biosphere seemed to be zero balanced, different from the inventory report [28]. The regional scale observation networks have been established in Switzerland and Paris, France, in order to assist regional greenhouse gas emission estimation. As it becomes more important to secure enough observations for surface CO_2_ flux optimization, more Asian observations are necessary to be utilized in inverse modeling.

In this study, CO_2_ observation data from Anmyeon-do (AMY) and Gosan (GSN), located in the Korean Peninsula, are assimilated into the CarbonTracker (CT), and the effect of the two observation datasets on Asian surface CO_2_ flux estimation is investigated. AMY and GSN sites have accumulated observation data over approximately more than 9 years, which could provide flux information over the Korean Peninsula and its surrounding Asian regions. Model and observations used are introduced in section 2. In section 3, the estimated CO_2_ fluxes in Asia from the 9-year (2003–2011) assimilation experiments using two additional observation datasets and the effects of assimilating AMY and GSN data into the inverse modeling are discussed. Finally, section 4 presents the summary and conclusions.

## 2. Methodology

### 2.1 Inverse modeling system

CT is a global scale inverse model [29] developed to estimate the surface CO_2_ fluxes using CO_2_ observations as a constraint. To assimilate CO_2_ mole fraction observations, CT adopts an ensemble Kalman filter (EnKF) DA method. Because the number of observation sites is too sparse to cover the whole globe, prior flux information needs to be given in advance. First guess of CO_2_ flux is presented as a combination of four different prior flux information as below:

$$F(x,y,t)=\lambda_{r}\cdot F_{bio}(x,y,t)+\lambda_{r}\cdot F_{ocn}(x,y,t)+F_{fire}(x,y,t)+F_{ff}(x,y,t),$$ (1)

where F_bio_, F_ocn_, F_fire_ and F_ff_ represent CO_2_ flux from terrestrial ecosystem activity (CASA GFED v3.1: [30, 31]), atmosphere-ocean CO_2_ exchange [32], biomass burning from the forest fire [30, 31], and fossil fuel combustion (Carbon Dioxide Information and Analysis Center [CDIAC] database: [33]), respectively. Since CO_2_ emissions from fossil fuel use and forest fire are prescribed, it is important to find precise biosphere flux and ocean flux estimates to optimize the total CO_2_ fluxes. In CT, scaling factor λ_r_ is updated through DA and used for optimizing the two CO_2_ flux components (i.e., biosphere and ocean fluxes). Each scaling factor corresponds to the specific ecosystem called ecoregion. The 126 ecoregions in the land among 209 ecoregions that combines Transcom regions (i.e., 11 regions) and ecosystem types (i.e., 19 types), and the 30 ecoregions in the ocean [34] are paired with scaling factors and those scaling factors update the flux values. These total 156 ecoregions are shown in CarbonTracker website (https://gml.noaa.gov/ccgg/carbontracker/CT2013B_doc.pdf). Among 209 ecoregions mentioned above, the unrealistic combinations of Transcom regions and ecosystem types (e.g., mangrove in the Eurasia Boreal) are not included in the 126 ecoregions in the land.

Transport Model 5 (TM5: [35]), an offline atmospheric chemical transport model, converts surface CO_2_ flux from Eq (1) into model CO_2_ concentration (i.e., mole fraction). TM5 then calculates advection, convection, and vertical diffusion of CO_2_ using ERA-Interim reanalysis data from the European Centre for Medium-Range Weather Forecasts (ECMWF). TM5 works as an observation operator calculating model CO_2_ concentration corresponding to the observed CO_2_ concentration at the time and space of which observation occurs.

The EnKF scheme used in CT is the ensemble square root Kalman filter (EnSRF) adopted from [36]. EnSRF separately updates ensemble mean and ensemble perturbation, as follows:

$${\bar{x}}^{a}={\bar{x}}^{b}+K(y^{o}‐H{\bar{x}}^{b}),$$ (2)

$${x^{\prime }}_{i}^{a}={x^{\prime }}_{i}^{b}‐\tilde{k}H{x^{\prime }}_{i}^{b},$$ (3)

where $\bar{x}$ is the ensemble mean of the state vector **x**, which is scaling factor being updated in the DA system in this study. Subscripts a and b are analysis and background, respectively, and **y**^O^ is the observation vector. **H** is a linear observation operator projecting the model state vector onto the observation space, and the TM5 works as **H** in CT. **K** and $\tilde{k}$ are Kalman gain and reduced Kalman gain, respectively.

$$K=(P^{b}H^{T}){\left(HP^{b}H^{T}+R\right)}^{‐1},$$ (4)

$$\tilde{k}=\alpha \cdot K,$$ (5)

where $\alpha ={\left(1+\sqrt{\frac{R}{HP^{b}H^{T}+R}}\right)}^{-1}$, **P**^b^ is the model’s background error covariance, and **R** is the observation error covariance. **P**^b^**H**^T^ and **HP**^b^**H**^T^ is calculated using the equations below:

$$P^{b}H^{T}\approx \frac{1}{N‐1}\left(x_{1}^{\prime },x_{2}^{\prime },\ldots ,x_{N}^{\prime }\right)\times {\left(Hx_{1}^{\prime },Hx_{2}^{\prime },\ldots ,Hx_{N}^{\prime }\right)}^{T},$$ (6)

$$HP^{b}H^{T}\approx \frac{1}{N-1}\left(Hx_{1}^{\prime },Hx_{2}^{\prime },\ldots ,Hx_{N}^{\prime }\right)\times {\left(Hx_{1}^{\prime },Hx_{2}^{\prime },\ldots ,Hx_{N}^{\prime }\right)}^{T},$$ (7)

where N is number of ensemble members.

It is necessary to prevent the sampling error amplification by limited ensemble members. Covariance localization technique [37] is conducted in CT to exclude the effect of the remote observations to the surface CO_2_ flux estimation, as the remote observations from the flux location are barely correlated with the flux concerned. Since no physical relationship exists between scaling factors, the correlations are calculated between scaling factor deviations of ensemble members and corresponding modeled flux deviations. If the correlation values fail the significance test, the scaling factor is not updated. Marine Boundary Layer (MBL) observations are exempt for the localization as MBL sites captures the flux signal from a distance [34].

On each week of the simulation period, the EnKF assimilates observations from the most recent week to update the scaling factors of the five weeks including the past four weeks as well as the most recent week. As [38] denoted, the time lag scheme helps to consider that the observations can contain the signal of sources or sinks away from the observing sites. Previous studies using a five-week time lag [7, 29, 39–41] showed that the five-week time lag is appropriate to optimize the surface CO_2_ flux in North America, Europe, and Asia.

In CT, the scaling factor for the upcoming analysis week is predicted by a simple model as follows:

$$\lambda_{t}^{b}=\frac{\left(\lambda_{t‐2}^{a}{+\lambda }_{t‐1}^{a}{+\lambda }^{p}\right)}{3},$$ (8)

where $\lambda_{t}^{b}$ is a prior scaling factor for the upcoming analysis week t; $\lambda_{t‐2}^{a}$ and $\lambda_{t‐1}^{a}$ are posterior scaling factors of week t-2 and t-1, respectively. λ^p^ is a fixed value 1, so that the scaling factor returns to 1 when there are no assimilated observations.

### 2.2 CO_2_ observation

In this study, two surface CO_2_ mole fraction observation datasets from AMY (https://gaw.kishou.go.jp/search/file/0039-2014-1001-01-01-9999) and GSN (https://gaw.kishou.go.jp/search/file/0052-2025-1001-01-01-9999) sites in the Korean Peninsula are additional observations that are assimilated in CT. AMY station has been operated by Korea Meteorological Administration (KMA) since 1998 and a year later has been designated as a regional global atmospheric watch (GAW) station. GSN station has started observation in 2002 by National Institute of Environmental Research (NIER), and KMA has taken over the operation since 2012 with new acronym JGS (Jeju Gosan). AMY and GSN data are obtained relatively remote from anthropogenic sources such as factories or residential area, which are appropriate to represent the CO_2_ concentration in Northeast Asia region. The method used for observing the two datasets is non-dispersive infrared analyzer (NDIR), which is able to log quasi-continuous CO_2_ concentration.

In this study, AMY and GSN data are assimilated together with other observation datasets from Observation Package (ObsPack) product in CT. The ObsPack product, provided by National Oceanic and Atmospheric Administration (NOAA) Earth System Research Laboratory (ESRL) [42], is a collection of CO_2_ observations around the world. Diverse research institutes including NOAA, the Commonwealth Scientific and Industrial Research Organization (CSIRO), the National Center for Atmospheric Research (NCAR), and Environment and Climate Change Canada (ECCC) have provided observed data for ObsPack production. Most ObsPack data are obtained by averaging observed values between 12–16 local standard time (LST) since the TM5 model shows good performance in simulating well-mixed atmospheric layer of daytime. For observation sites located at the mountaintops, observations between 00–04 LST are averaged because there is less chance of local biogenic or anthropogenic CO_2_ inflow from the downslope during the nighttime [34]. Daily mean AMY and GSN data are also obtained by averaging 12–16 LST data, following the ObsPack data.

Model-data mismatch (MDM) (i.e., observation error) for ObsPack is prescribed based on observation type and geographic characteristics in CT. When assimilated, the observation error for two datasets needs to be prescribed. MDM of both AMY and GSN is set to 3 ppm based on several verifications conducted in [43]. Beside AMY and GSN, Tae-ahn Peninsula (TAP) data are already included in the ObsPack. Note that TAP’s MDM is 5 ppm, following [41].

**Fig 1:**
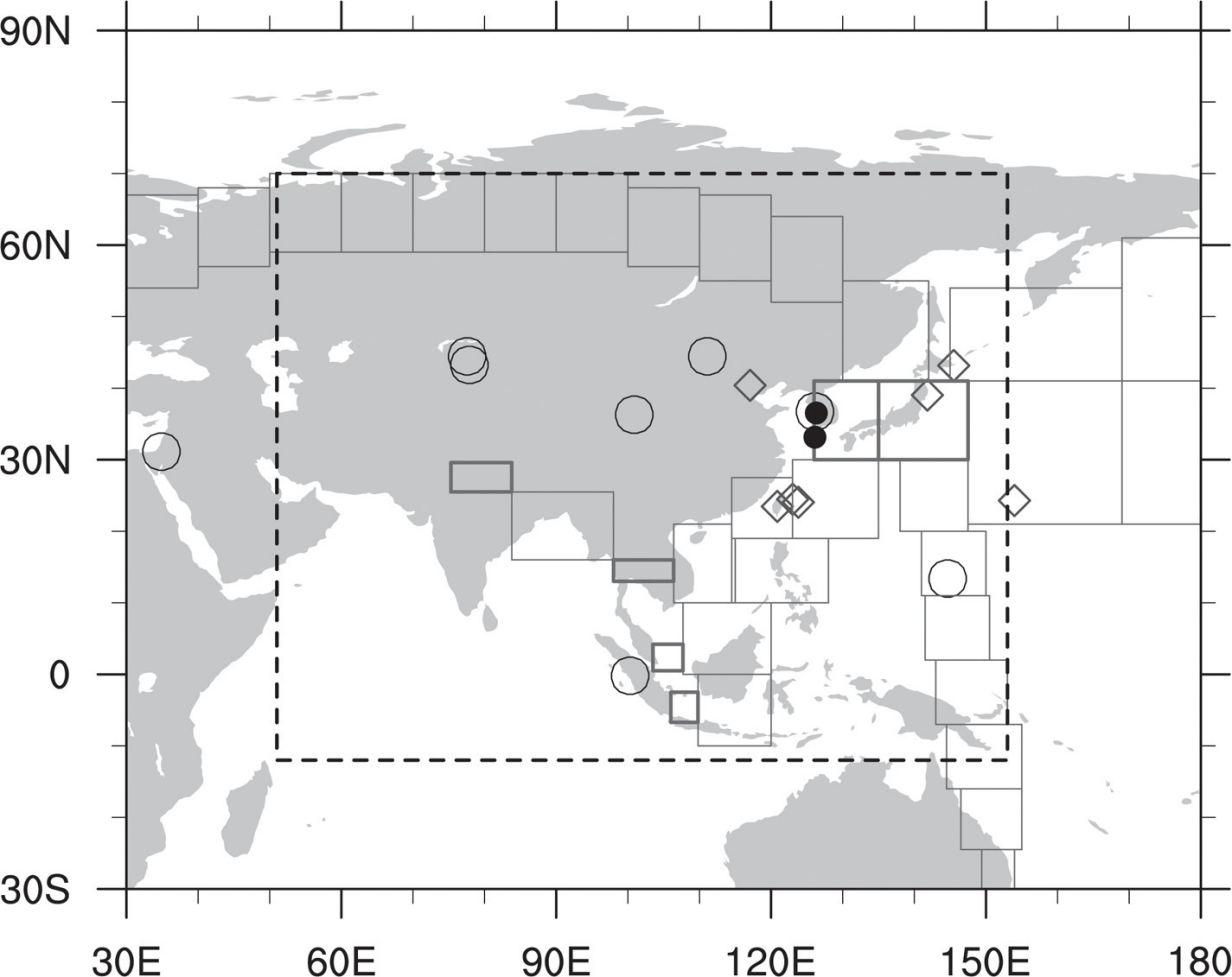
Observation sites in Asia, used either in data assimilation (○: NOAA ObsPack sites in Asia; ●: AMY and GSN) or in verification (◇: Observation sites of JMA, NIES, and ESRL). CONTRAIL observations are marked as squares (thick line: locations of ascending/descending mode; thin line: level mode). The dashed box represents the nested domain in TM5 transport model.

For verification of the results, independent CO_2_ observations in Asia that are not assimilated in CT are used. Those independent observations are aircraft observation data from the National Institute for Environmental Studies (NIES) Japan, called The Comprehensive Observation Network for Trace gases by Airliners (CONTRAIL) (http://doi.org/10.17595/20180208.001; [44, 45]), and surface observations from World Data Centre for Greenhouse Gases (WDCGG, https://gaw.kishou.go.jp/). Tables 1 and 2 and Fig 1 present information of the observations in Asia, used for assimilation and verification in this study. Note that only Asian ObsPack stations are depicted here. The CONTRAIL data used for verification shown in Fig 1 is from Nov. 2005 to Dec. 2011. As Fig 1, all figures including map image in this study were produced using NCL [46].

**Table 1:** Information of observation sites in Asia used for assimilation in this study.

Site	Location	Latitude	Longitude	Height [m]	Laboratory	Data period	MDM [ppm]
AMY	Anmyeon-do, South Korea	36.53°N	126.32°E	2002.01~2004.06: 57	KMA/ESRL	2002.01~	3
				2004.07~2011.12: 87		2011.12	
GSN	Gosan, South Korea	33.15°N	126.12°E	72	NIER	2002.01~	3
						2011.05	
TAP	Tae-ahn Peninsula, South Korea	36.37°N	136.13°E	20	ESRL	2002.01~	5
						2011.12	
WLG	Mt. Waliguan, China	36.29°N	100.9°E	3810	CMA/ESRL	2002.01~	1.5
						2011.12	
UUM	Ulaan Uul, Mongolia	44.45°N	111.10°E	914	ESRL	2002.01~	2.5
						2011.12	
GMI	Mariana Islands, Guam	13.38°N	144.65°E	2	ESRL	2002.01~	1.5
						2011.12	
BKT	Bukit Kototabang, Indonesia	0.20°S	100.32° E	864	ESRL	2004.01~	7.5
						2011.12	
KZD	Sary Taukum, Kazakhstan	44.45°N	77.57°E	412	ESRL	2002.01~	2.5
						2009.08	
KZM	Assy Plateau, Kazakhstan	43.25°N	77.88°E	2519	ESRL	2002.01~	2.5
						2009.08	
WIS	Sde Boker, Israel	31.13°N	34.88°E	400	ESRL	2002.01~	2.5
						2011.12	

Note that AMY has two different observation heights for the two periods.

**Table 2:** Information of observation sites in Asia used for independent verification in this study.

Site	Location	Latitude	Longitude	Height [m]	Laboratory	Data period
MNM	Minamitorishima, Japan	24.29°N	153.98°E	8	JMA	2002.01~
						2011.12
RYO	Ryori, Japan	39.03°N	141.82°E	260	JMA	2002.01~
						2011.12
YON	Yonagunijima, Japan	24.47°N	123.02°E	30	JMA	2002.01~
						2011.12
COI	Cape Ochiishi, Japan	43.16°N	145.5°E	96	NIES	2002.01~
						2010.12
HAT	Hateruma Island, Japan	24.05°N	123.81°E	46.5	NIES	2002.01~
						2010.12
LLN	Lulin, Taiwan	23.47°N	120.87°E	2862	ESRL	2006.08~
						2011.12
SDZ	Shangdianzi, China	40.39°N	117.07°E	287	CMA/ESRL	2009.09~
						2011.12

### 2.3 Experimental framework

CT2013B version is used in this study, which is able to simulate the surface CO_2_ flux from 2000 to 2012. Two experiments are conducted to investigate the impact of AMY and GSN observations on surface CO_2_ flux estimation. EXP1 experiment assimilates all available observations (AMY, GSN observations, and ObsPack datasets), while CNTL assimilates only ObsPack data. The TM5 model runs on a two-way nested grid with a 3°⨯2° outer domain on the globe and a 1°⨯1° nesting domain centered on Asia (Fig 1). The experimental period is from 2002 to 2011 because both AMY and GSN datasets exist almost completely for this period. The experimental results are analyzed for 9 years from 2003 to 2011 except for the first year (i.e., 2002) as a spin-up. More details about the experimental settings are summarized in Table 3.

**Table 3:** Experimental framework for estimating surface CO_2_ flux in Asia.

Experiment name		CNTL	EXP1
Common observation dataset		ObsPack CO_2_ PROTOTYPE v1.0.4b	
		(2014-2-13 released)	
Additional observation site		-	AMY(3), GSN(3)
(MDM [ppm])			
Model domain	3°⨯2°	Globe	
	1°⨯1°	51°~153°E, 12°S~70°N	
Experimental period		2002. 1. 1 ~ 2011. 12. 31 (2002: spin-up)	
Weeks of lag		5	
Number of ensemble members		150	

## 3. Results

### 3.1. Characteristics of surface CO_2_ flux in Asia

#### 3.1.1 9-year average surface CO_2_ flux distribution

**Fig 2:**
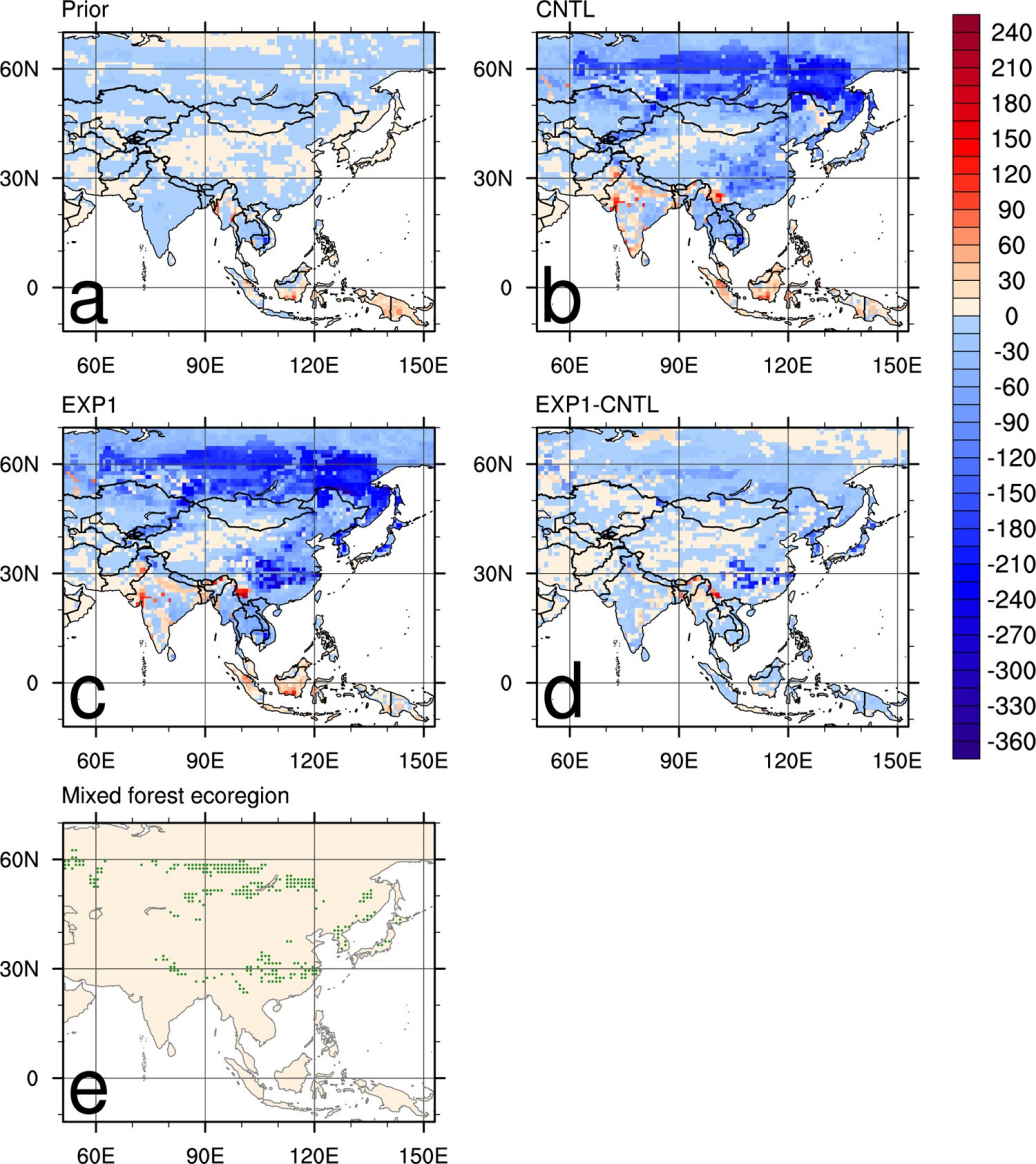
Average biosphere fluxes (g C m^-2^ yr ^-1^) for 2003–2011 period over the nested Asian domain: (a) prior, (b) CNTL, (c) EXP1, and (d) difference between EXP1 and CNTL. (e) Spatial distribution of the “Mixed forest” ecoregion in Asia, where AMY site is located.

Fig 2 shows the 9-year average surface CO_2_ flux distribution within the nested domain shown in Fig 1. Fig 2A shows the surface CO_2_ flux calculated by prior fluxes (i.e., with no DA), Fig 2B and 2C are the CNTL and EXP1 results, Fig 2D is the average flux difference between EXP1 and CNTL, and Fig 2E shows the spatial distribution of the “Mixed forest” ecoregion in Asia where the AMY site is located. DA enhances the absorption and emission of surface CO_2_ fluxes compared with prior fluxes (Fig 2A–2C). In particular, there are strong CO_2_ flux absorptions in the Indochina Peninsula, inland China, the Korean Peninsula, Japan, and southern Siberia, and strong CO_2_ flux emissions in inland India and a small part of southern China (Fig 2B and 2C). The average flux distributions of CNTL and EXP1 show generally similar patterns (Fig 2B and 2C), but EXP1 shows more absorption than CNTL does in inland southern China, the Korean Peninsula, and Japan (Fig 2D). Locations such as the border region between northern Thailand and China represent greater emissions in EXP1 than in CNTL (Fig 2D). Mixed forest areas coincide well with the areas where the DA effects are obvious (Fig 2B, 2C and 2E), which is due to background error covariance in the EnKF in CT. As each scaling factor is assigned to respective ecoregions, the background error covariance matrix shows correlations between ecoregions in different Transcom regions. Since the dynamical model in Eq (8) does not include an error term, the background error covariance is set to a prior covariance structure and not predicted with the dynamical model [29]. According to [34], the same ecoregions among five different Transcom regions (North American Boreal, North American Temperate, Eurasia Boreal (EB), Eurasia Temperate (ET), and Europe) have correlations although those correlations become small for distant ecoregions. These correlations allow observations in a certain ecoregion to update scaling factors connected to the same ecoregion concerned, through the DA. It explains how AMY data affects more on the specific areas.

#### 3.1.2 Annual and average surface CO_2_ flux

**Fig 3:**
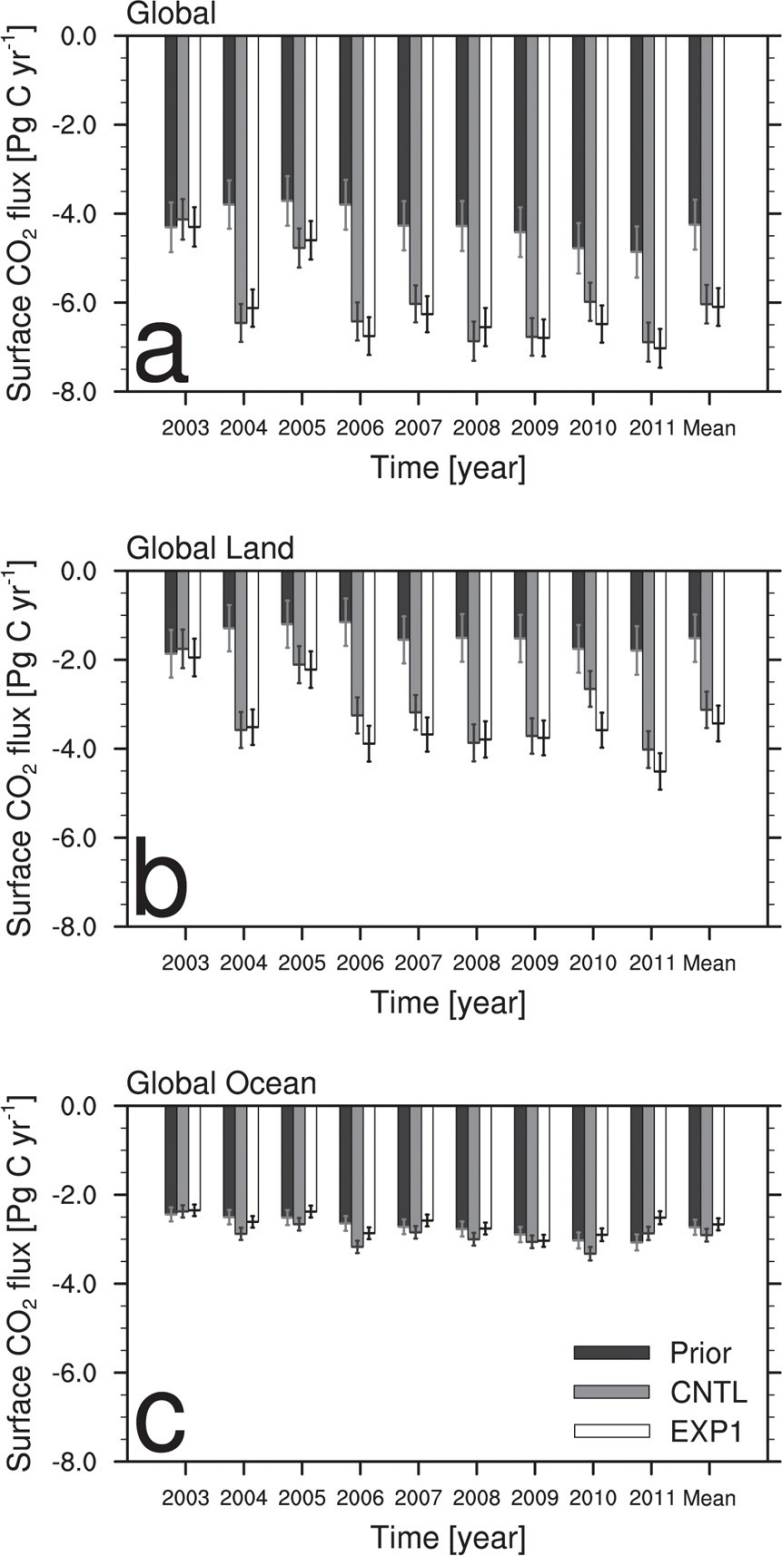
Annual and average biosphere and ocean CO_2_ fluxes (Pg C yr^-1^) from the prior (black), CNTL (gray), and EXP1 (white) with their uncertainties aggregated over the (a) whole globe, (b) land, and (c) ocean.

Fig 3 shows annual and average surface CO_2_ fluxes on the globe, the land, and the ocean. Compared to prior flux, global CO_2_ flux uptake by land vegetation and ocean in CNTL and EXP1 is approximately 2 Pg C yr^-1^ greater (Fig 3A). Most of this CO_2_ uptake difference between the experiments and prior flux comes from the CO_2_ absorption by the terrestrial vegetation (Fig 3B), while CO_2_ flux absorptions from the ocean in the CNTL and EXP1 are only slightly different from the prior flux (Fig 3C). When comparing CNTL and EXP1 results, EXP1 shows slightly more (less) biogenic (oceanic) CO_2_ flux absorption than CNTL does. The interannual variation of CNTL and EXP1 during the analysis period is very similar, indicating that the two Korean observation datasets assimilated in CT did not interrupt the consistency in the global surface CO_2_ flux variability. The prior fluxes show the greatest uncertainties on the globe, the land, and the ocean, followed by CNTL and EXP1 (Fig 3A–3C). The average uncertainty of the prior flux for 9 years on the globe decreases by 22.5% in CNTL and 24.2% in EXP1 (Fig 3A). The decreases of uncertainties in CNTL and EXP1 compared to the prior flux uncertainty are greater on the land than the ocean (Fig 3B and 3C).

**Fig 4:**
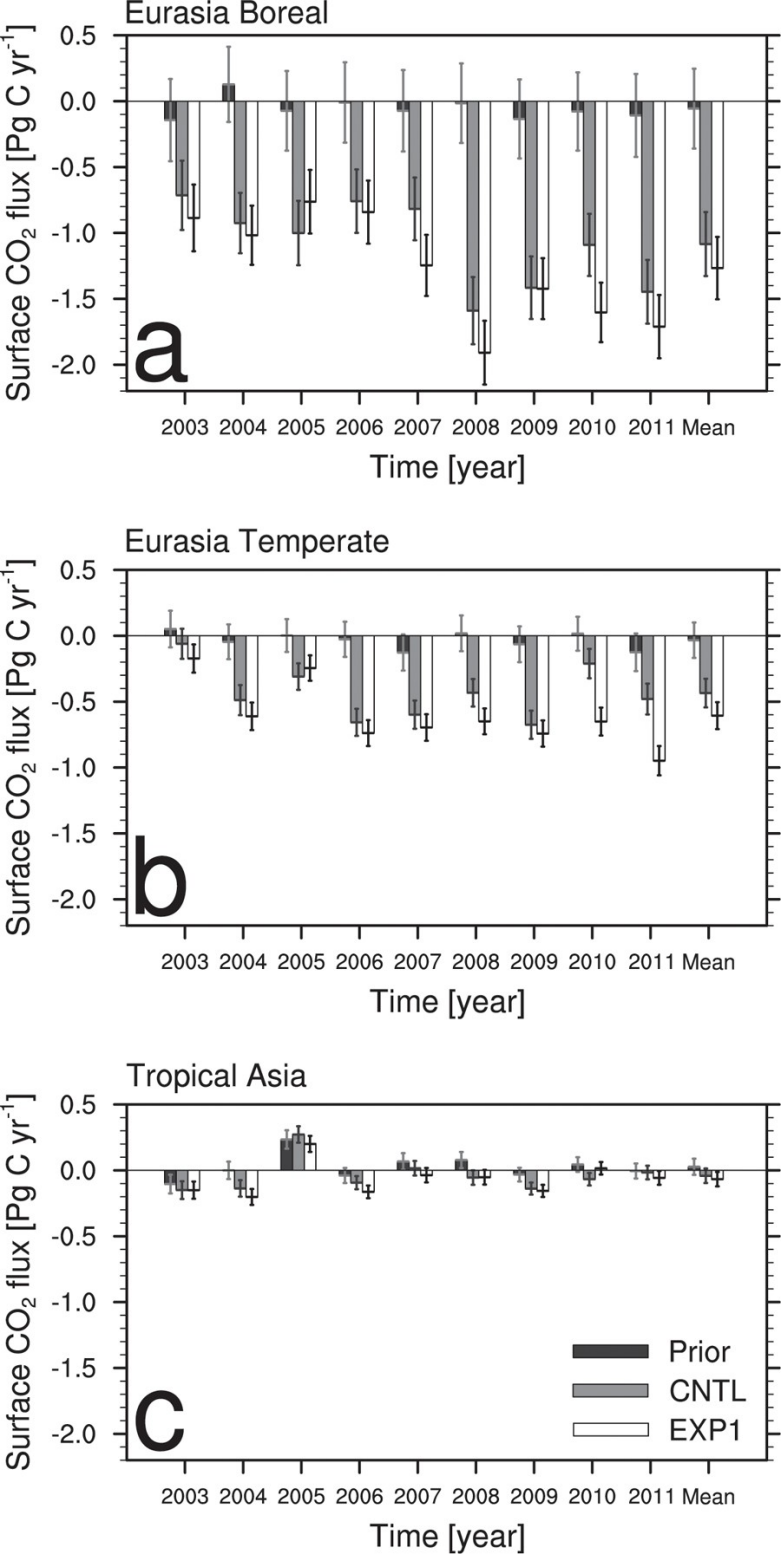
Annual and average biosphere CO_2_ fluxes (Pg C yr^-1^) from the prior (black), CNTL (gray), and EXP1 (white) with their uncertainties aggregated over the (a) Eurasia Boreal, (b) Eurasia Temperate, and (c) Tropical Asia.

Fig 4 is the same as Fig 3 but for the three Transcom regions in Asia. In the EB and ET regions, the surface CO_2_ fluxes from the prior flux are very small, while CNTL and EXP1 result in absorbing large amounts of surface CO_2_ fluxes. Compared to CNTL, EXP1 generally estimates more biogenic CO_2_ flux absorption for every Transcom regions in Asia. CNTL and EXP1 estimate -1.08 Pg C yr^-1^ and -1.27 Pg C yr^-1^ of CO_2_ flux for EB (Fig 4A), while -0.43 Pg C yr^-1^ and -0.61 Pg C yr^-1^ of CO_2_ flux for ET (Fig 4B). In particular, compared to CNTL, EXP1 shows larger biogenic surface CO_2_ flux absorption in ET since the added two Korean CO_2_ observation sites are located in ET. The biogenic CO_2_ flux absorption in EB is also affected by the two observation datasets in ET owing to the background error covariance structure described in section 3.1.1. Assimilation of AMY and GSN datasets results in negative CO_2_ fluxes greater in Asia, which implies the possibility of the enhanced CO_2_ absorption as well as the weakened CO_2_ respiration. The prior fluxes show the greatest uncertainties in EB, ET, and Tropical Asia (TA), followed by CNTL and EXP1 (Fig 4A–4C). The average uncertainty of the prior flux for 9 years in EB decreases by 19.8% in CNTL and 21.8% in EXP1 (Fig 4A), and that in ET decreases by 18.7% in CNTL and 23.9% in EXP1 (Fig 4B). In TA, CNTL and EXP1 show very similar uncertainties (Fig 4C).

In EXP1, the EB region has the lowest flux absorption in 2005 and 2006 and the greatest flux absorption in 2008 and 2011 (Fig 4A). The ET has the lowest CO_2_ uptake in 2003 and 2005 and the greatest in 2011 (Fig 4B). This interannual variability of biogenic CO_2_ flux seems to be affected by the climate events. It is known that El Nino enhances the CO_2_ sources while La Nina intensifies the CO_2_ sinks [47, 48]. Based on the ENSO ONI index from NCEP (https://origin.cpc.ncep.noaa.gov/products/analysis_monitoring/ensostuff/ONI_v5.php), the strong La Nina events occurred during 2007–2008 and 2010–2011, and the biogenic CO_2_ flux absorption estimated from the CT increased at the same period. Meanwhile, weak El Nino events occurred during 2004–2005 and late 2009, and the biogenic CO_2_ flux absorption from CT weakened during that period. Additionally, extreme drought conditions occurred in 2003 in all of the northern midlatitudes [49] result in reduced uptake of CO_2_ [6]. Therefore, assimilating CO_2_ observation datasets in Korea reflects the climate effect on the surface CO_2_ exchange in EB and ET (Fig 4A and 4B). TA has very small CO_2_ uptake and emission of less than 0.3 Pg C yr^-1^, irrespective of the experiments (Fig 4C). Surface CO_2_ flux estimates over the TA region have been known to have high uncertainty because there are little observations for the inverse modeling to represent the signal of source and sink [39, 50, 51]. [52, 53] showed that the near-neutral CO_2_ flux in tropical region is due to the balance between the CO_2_ release from deforestation and the CO_2_ uptake by the intact tropical forests. [48] showed that the carbon budget in South Asia and Southeast Asia is close to neutral, with weak signs of carbon sink.

#### 3.1.3 Monthly and weekly aggregated surface CO_2_ flux

**Fig 5:**
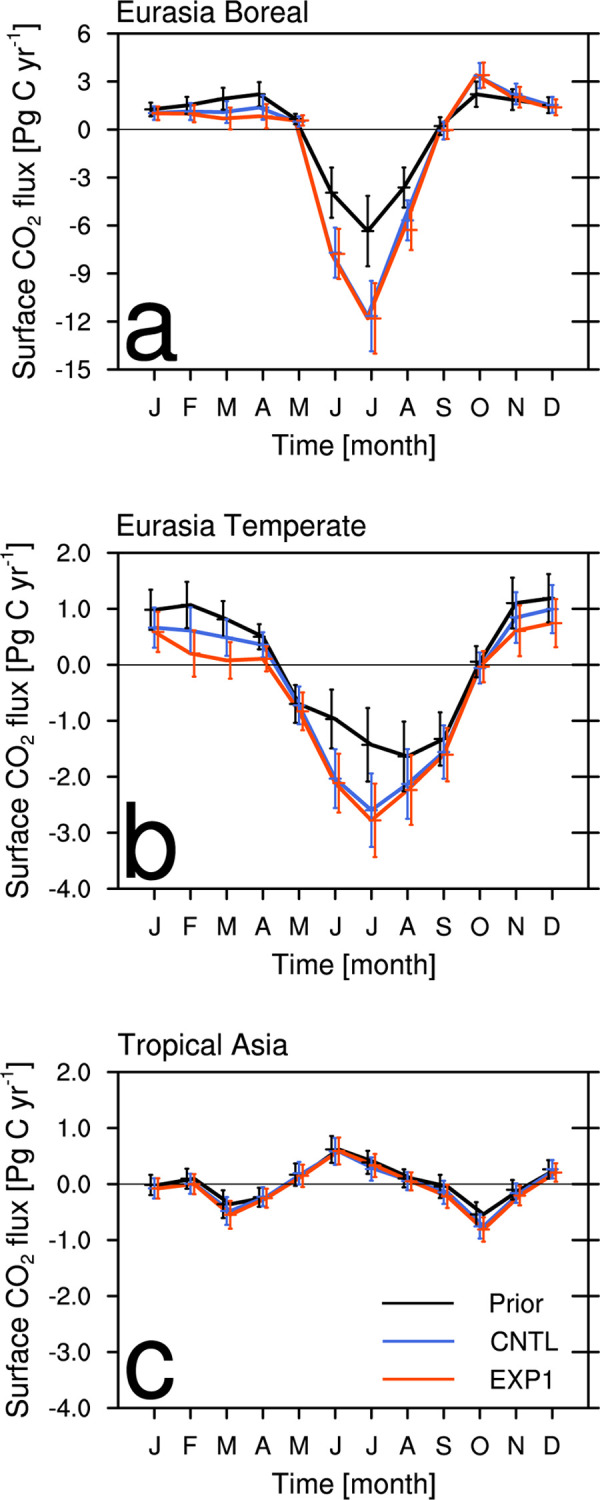
Monthly biosphere CO_2_ fluxes (Pg C yr^-1^) of the prior (black), CNTL (blue), and EXP1 (red), averaged for 2003–2011 period, with their uncertainties over the (a) Eurasia Boreal, (b) Eurasia Temperate, and (c) Tropical Asia.

Fig 5 shows the time series of monthly surface CO_2_ fluxes averaged in the analysis period (i.e., 2003–2011) for the individual Transcom regions in Asia. In both CNTL and EXP1, a distinct seasonal variation pattern is found in the EB and ET regions (Fig 5A and 5B), in which flux absorption occurs in summer and flux emission occurs from autumn to spring.

Over the EB region, where vegetation activity is very active, approximately -12 Pg C yr^-1^ is estimated to be absorbed to the surface every summer with a large surface CO_2_ flux uncertainty (Fig 5A). Compared to CNTL, CO_2_ flux emission in spring in EXP1 decreases in the EB region. The ET region shows large difference between CNTL and EXP1 (Fig 5B). Compared to CNTL, EXP1 shows stronger flux absorption in the summer and weakened flux emission in winter and spring. In particular, CO_2_ flux emission in spring in EXP1 is reduced to less than half of CNTL. The uncertainties of surface CO_2_ flux estimation in CNTL and EXP1 are the greatest in summer. In the TA region, there is little difference between CNTL and EXP1 and there is no distinct seasonal variation although there are CO_2_ flux absorption in spring and fall and release in summer (Fig 5C).

Therefore, the assimilation of AMY and GSN observations in CT enhances the monthly surface CO_2_ flux absorption in summer in Asia region (especially EB and ET), and decreases the CO_2_ emission in spring and winter season. The seasonal surface CO_2_ fluxes would be changed if the seasonal and diurnal variations of MDM are considered. The effect of MDM variations in estimating the surface CO_2_ fluxes over Asia would be a future study.

**Fig 6:**
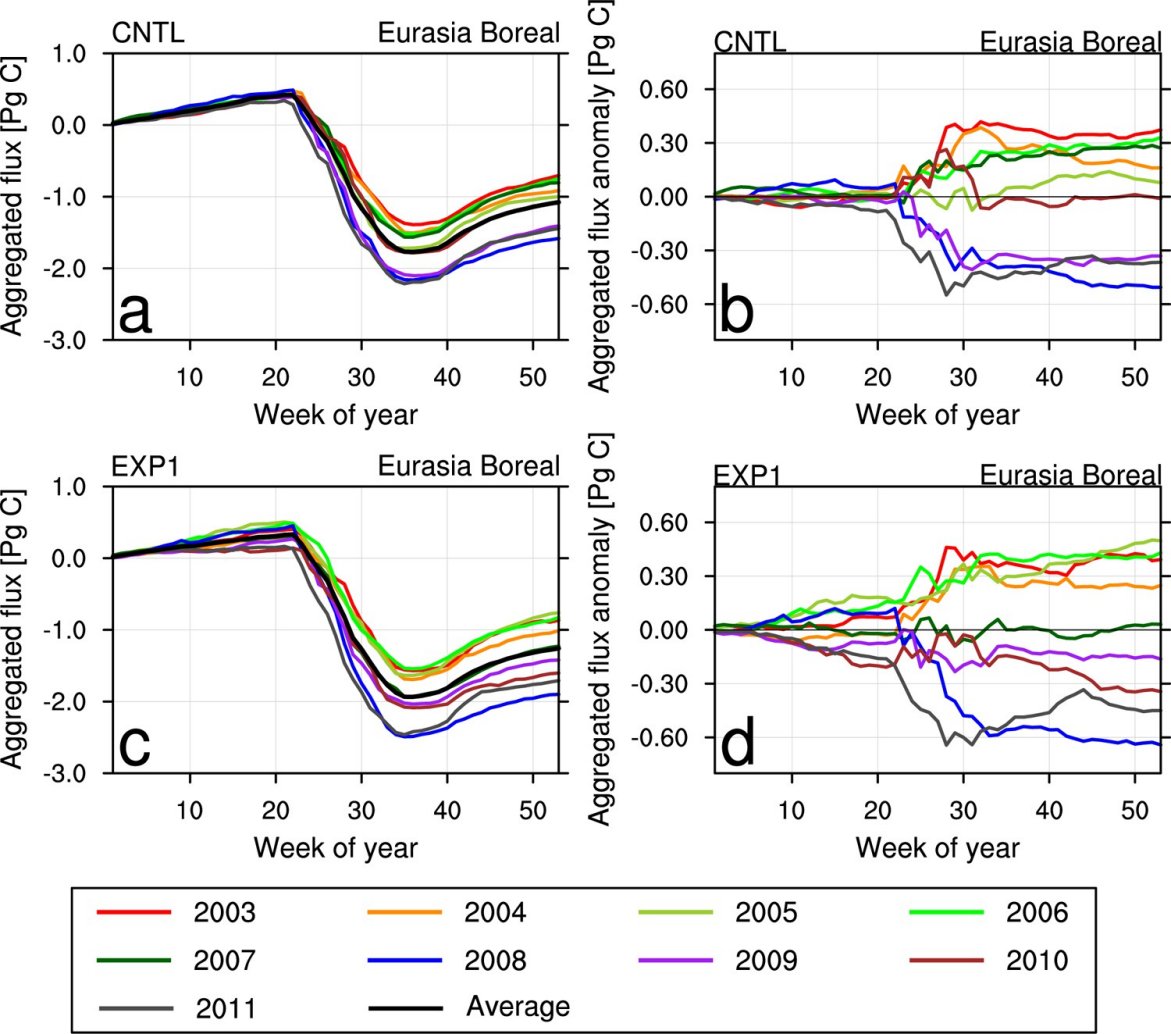
Weekly cumulative biosphere CO_2_ fluxes (Pg C) of each year over Eurasia Boreal region: (a) CNTL and (c) EXP1. Weekly cumulative biosphere CO_2_ flux differences (Pg C) in each year from the 9-year average for 2003–2011 period over Eurasia Boreal region: (b) CNTL and (d) EXP1.

**Fig 7:**
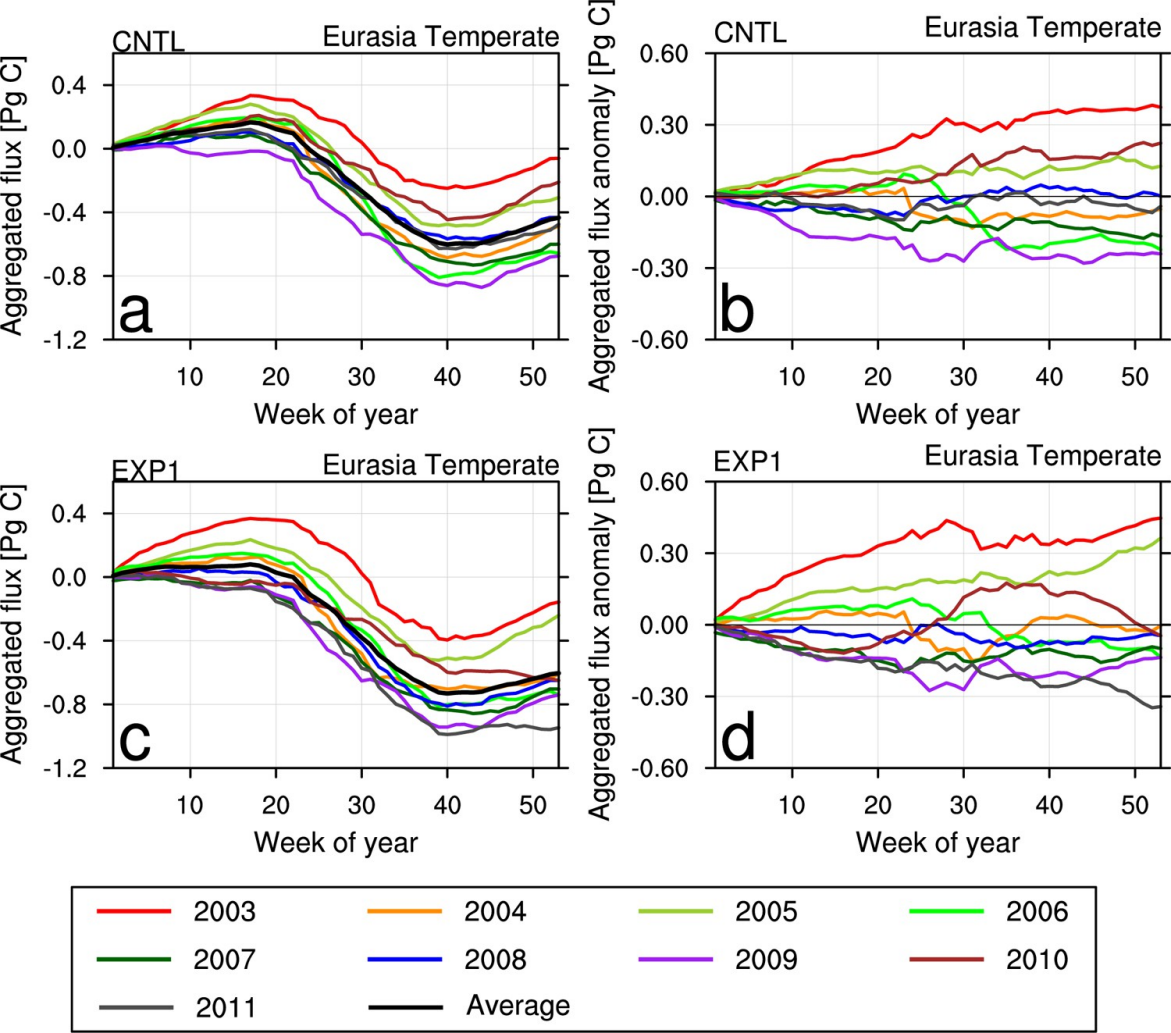
Same with Fig 6 except for Eurasia Temperate (ET) region.

Figs 6 and 7 show the weekly cumulative fluxes of each year and their differences from 9-year average values calculated for EB and ET, respectively. The EB region shows seasonal variation every year, absorbing CO_2_ strongly in summer and emitting CO_2_ in spring and winter (Fig 6A and 6C). CNTL shows a decrease in summer CO_2_ flux absorption in 2003, 2004, 2006, and 2007, whereas shows strong above average CO_2_ flux absorption in summer of 2008, 2009, and 2011 (Fig 6B). In EXP1, the flux absorption is much more active during the whole analysis period since averaged weekly cumulative flux is shifted to the negative direction (Fig 6C). From 2007 to 2011, the flux absorption in EXP1 is similar to or greater than the 9-year averaged flux (Fig 6D).

The ET region shows seasonal variation similar to the EB region, but its magnitude decreases by half (Fig 7A and 7C). In CNTL, the flux absorption is weaker than the 9-year averaged flux absorption in 2003, 2005, and 2010, whereas the flux absorption is stronger and emission is weaker in 2006, 2007, and 2009 (Fig 7B). The average cumulative CO_2_ flux for EXP1 is lower than the CNTL, indicating EXP1 uptakes more CO_2_ flux than CNTL does (Fig 7D). In EXP1, strong spring CO_2_ uptake occurred in 2007 and from 2009 to 2011, which made the average cumulative flux in spring period close to zero. In 2010, CO_2_ uptake in EXP1 decreased from summer to fall, which is similar to the CNTL result, but EXP1 showed more CO_2_ flux absorption in spring and early winter.

Overall, more weekly cumulative CO_2_ absorption is simulated for the terrestrial biosphere in Asia, and the flux differences are more diverse when assimilating the two Korean observation datasets in CT.

### 3.2 Verification with independent observations

#### 3.2.1 Verification with surface observations

Table 4 summarizes bias and RMSE of model CO_2_ concentrations with respect to observed CO_2_ concentrations, and correlation coefficient between model CO_2_ concentrations and observed CO_2_ concentrations, for seven independent surface CO_2_ observation sites in Asia. CNTL shows positive bias for every site except RYO, implying that the CNTL generally overestimates observed CO_2_ concentration at evaluation sites. EXP1 also shows positive bias for all sites, but their absolute values are smaller than those of CNTL except RYO, which indicates that EXP1 estimates more accurate CO_2_ concentrations than CNTL.

**Table 4:** Bias (ppm), RMSE (ppm), and correlation coefficient of model CO_2_ concentrations of CNTL and EXP1 with respect to observed CO_2_ concentrations at seven independent surface observation sites in Asia.

Site	Bias [ppm]		RMSE [ppm]		Correlation coefficient	
	CNTL	EXP1	CNTL	EXP1	CNTL	EXP1
COI	0.161	0.125	2.747	2.830	0.926	0.917
HAT	0.447	0.421	1.392	1.460	0.972	0.968
RYO	-0.082	0.107	2.916	3.386	0.919	0.889
MNM	0.600	0.592	0.959	0.970	0.992	0.991
YON	0.853	0.779	2.131	2.152	0.949	0.945
LLN	4.064	3.964	5.699	5.617	0.750	0.755
SDZ	1.059	0.861	7.285	6.535	0.645	0.701
Average	1.015	0.978	3.304	3.279	0.879	0.881

The monthly model CO_2_ concentrations in CNTL and EXP1 are mostly overestimated compared to the monthly observed CO_2_ concentrations and the biases are relatively smaller in winter than in summer, indicating better performance of CT in winter (not shown). EXP1 shows smaller biases than CNTL during November to April period except January.

The RMSE of CNTL is smaller than that of EXP1 for COI, HAT, RYO, MNM, and YON sites, which are located on islands or seaside. In contrast, the RMSE of EXP1 is smaller than that of CNTL for LLN and SDZ sites which are located inland. Averaging over all sites, the RMSE of EXP1 (3.279 ppm) is slightly less than that of CNTL (3.304 ppm). Pearson correlation coefficient shows similar results to the RMSE results. SDZ and LLN have higher correlation values in EXP1 than CNTL, while the other sites have higher correlation coefficient values in CNTL than EXP1.

The evaluation with the independent surface CO_2_ observations indicates that, by assimilating AMY and GSN site observations into CT, the bias of model CO_2_ concentration could be reduced and the model CO_2_ concentration with DA could be more accurate than that without DA, particularly over inland vegetation region. In terms of monthly verification, the bias of monthly model CO_2_ concentration could be reduced for the winter to early spring seasons, by assimilating AMY and GSN site observations into CT.

#### 3.2.2 Verification with CONTRAIL aircraft observations

**Fig 8:**
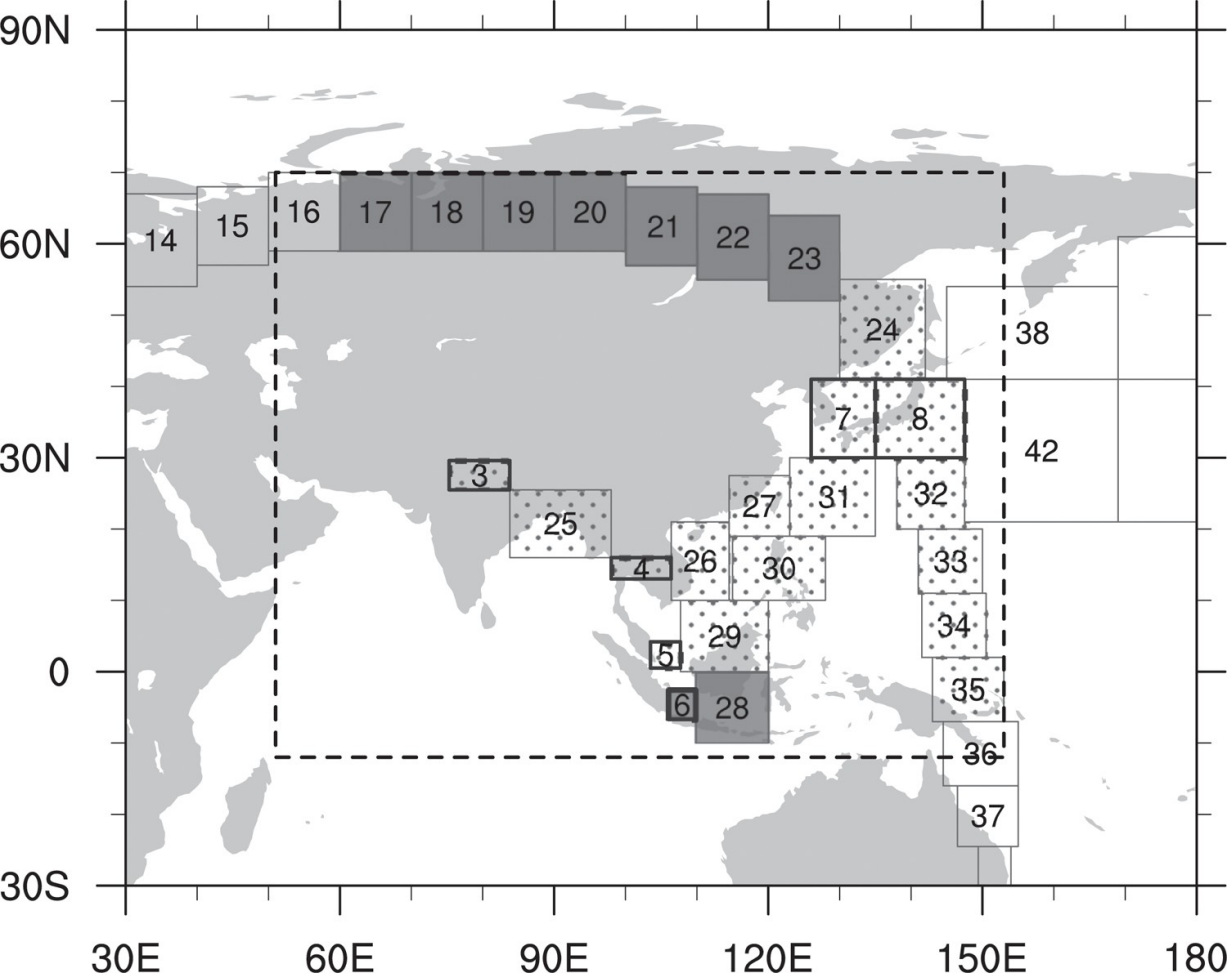
CONTRAIL bins filled with dark gray represent that RMSEs of CNTL are smaller than those of EXP1, while bins filled with dots represent that RMSEs of EXP1 are smaller than those of CNTL. The numbers are assigned on bins to identify the observational regions of CONTRAIL data.

In this section, the model CO_2_ concentrations for each experiment are verified with respect to the independent CONTRAIL observations, which are not assimilated in any of the experiments. CONTRAIL observations are categorized with two types of observation mode: ascending/descending mode and level mode (Fig 8). The vertical observation (ascending/descending mode) is conducted while ascending from/descending to the airport. Bin numbers 3 to 8 represent the ascending/descending mode where the observations are conducted at four different levels (level 1: 625–575 hPa, level 2: 525–475 hPa, level 3: 425–375 hPa, and level 4: 275–225 hPa). The 6 bins drawn with thick boundaries represent ascending/descending mode, and their locations are New Delhi (bin 3), Bangkok (bin 4), Singapore (bin 5), Jakarta (bin 6), Incheon (bin 7), and Tokyo (bin 8). The other bins (bin numbers 17 to 35) contain the observations from the upper atmospheric layer of 275–225 hPa (level mode). The observations in the stratosphere are excluded from the analysis. The method of separating CONTRAIL data into several bins follows [7].

Table 5 shows RMSE of model CO_2_ concentrations for each experiment with respect to the CONTRAIL observations. The average RMSEs for total CONTRAIL data are smaller in EXP1 (1.506 ppm) than in CNTL (1.545 ppm), and those for the ascending/descending mode are also smaller in EXP1 (1.581 ppm) than in CNTL (1.643 ppm). At bins 3, 4, 7 and 8, EXP1 shows smaller RMSEs than CNTL does at each level. At bin 5, EXP1 shows smaller RMSE than CNTL does only at level 4. At bin 6, CNTL shows smaller RMSEs than EXP1 does at level 1, 2 and 4. In case of the level mode, the RMSE of EXP1 (1.414 ppm) is also smaller than that of CNTL (1.424 ppm).

**Table 5:** RMSE (ppm) of model CO_2_ concentrations of CNTL and EXP1 with respect to observed CO_2_ concentrations at the CONTRAIL bins in the nested domain.

Ascending/Descending mode				Level mode		
Bin	Level	CNTL	EXP1	Bin	CNTL	EXP1
**3**	**1**	2.632	2.523	**17**	1.650	1.660
	**2**	2.573	2.447	**18**	1.683	1.717
	**3**	3.057	2.914	**19**	1.652	1.675
	**4**	3.065	2.936	**20**	1.658	1.691
**4**	**1**	1.321	1.270	**21**	1.707	1.713
	**2**	1.319	1.242	**22**	1.685	1.709
	**3**	1.319	1.253	**23**	1.757	1.777
	**4**	1.501	1.454	**24**	1.793	1.758
**5**	**1**	0.936	0.959	**25**	2.306	2.287
	**2**	0.956	0.976	**26**	1.134	1.111
	**3**	0.913	0.918	**27**	1.247	1.224
	**4**	0.906	0.886	**28**	1.125	1.137
**6**	**1**	0.835	0.851	**29**	0.847	0.838
	**2**	0.881	0.882	**30**	0.914	0.897
	**3**	0.879	0.878	**31**	1.322	1.288
	**4**	1.087	1.111	**32**	1.030	0.972
**7**	**1**	1.599	1.560	**33**	0.756	0.697
	**2**	1.637	1.571	**34**	0.673	0.637
	**3**	1.752	1.683	**35**	0.705	0.696
	**4**	1.638	1.570			
**8**	**1**	1.642	1.615	**Mode**	**CNTL**	**EXP1**
	**2**	1.564	1.504	**Ascending/Descending**	1.643	1.581
	**3**	1.575	1.497	**Level**	1.424	1.414
	**4**	1.649	1.591	**Total**	1.545	1.506

Bin numbers from 3 to 8 represent the ascending/descending mode, while bin numbers from 17 to 35 represent the level mode. The gray shaded table denotes the average RMSEs for each observation mode.

When considering the whole levels for the ascending/descending mode observation, the bins 3, 4, 5, 7 and 8 have the smaller RMSEs in EXP1, while the bin 6 has the smaller RMSE in CNTL (Fig 8). For the level mode observation, bins 24–27 and 29–35 have the smaller RMSEs in EXP1, and the other level mode bins (i.e., 17–23 and 28) have the smaller RMSEs in CNTL. The bins showing smaller RMSEs in EXP1 are located over ET regions including Korea and Japan, TA regions, and Northwest Pacific Ocean. The bins showing smaller RMSEs in CNTL are located over the Siberian regions (EB) and near the equator.

Overall, the evaluation with the independent CONTRAIL observations shows that assimilating additional Korean observations into the CT led to more accurate surface CO_2_ flux estimations over Asia.

### 3.3 Uncertainty reduction

**Fig 9:**
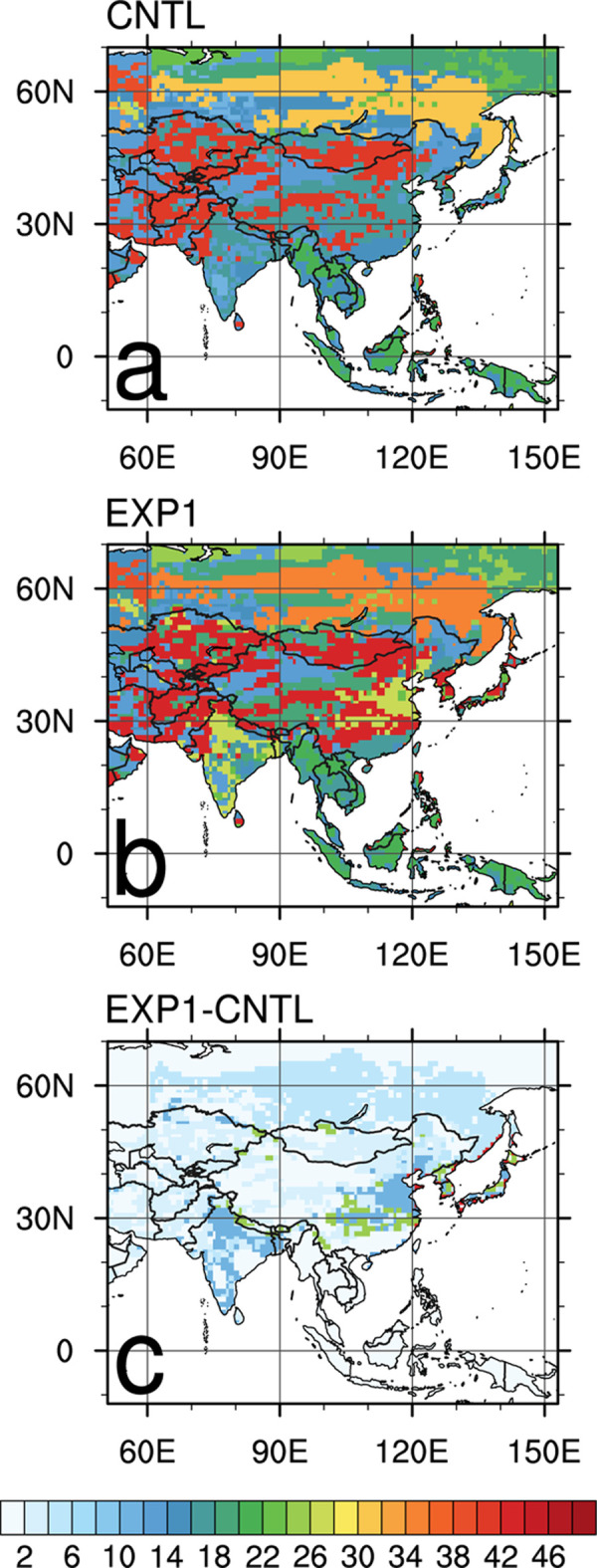
Average uncertainty reduction (%) for 2003–2011 period: (a) CNTL, (b) EXP1, and (c) the difference between EXP1 and CNTL.

Fig 9 shows the uncertainty reduction rate of each experiment for the estimated posterior surface CO_2_ flux compared to the prior surface CO_2_ flux, averaged over the analysis period. The area of maximum uncertainty reduction in CNTL appears on parts of inner China, Mongolia, and central Asia regions, showing approximately 40% of reduction. It is followed by Siberian region near 60°N latitude, showing approximately 28% of uncertainty reduction after the optimization (Fig 9A). For EXP1, the uncertainty reduction is approximately 59% on Eastern China, Korea, and Japan, where the location coincides with the mixed forest ecoregion of ET (Fig 9B). Uncertainty reduction rate around 60°N Siberian region is still relatively high in EXP1 compared to CNTL, nearly 35% of uncertainty decreases. Some parts of India show 27% uncertainty reduction in EXP1.

The difference of the uncertainty reduction between EXP1 and CNTL is shown in Fig 9C. Compared to CNTL, EXP1 shows more uncertainty reduction over Eastern China, Korea, Japan, and India, located in ET region. In Siberia, there is no distinct difference in uncertainty reduction between CNTL and EXP1. EB and TA regions also show little differences between EXP1 and CNTL. Therefore, the uncertainties in the estimated surface CO_2_ flux over ET are reduced by adding the two Korean observation datasets in CT.

### 3.4 Influence matrix

An influence matrix is used to measure the influence of assimilated observation data on the model result. [6, 41, 54] calculated the influence of surface CO_2_ observation data assimilated on the estimated surface CO_2_ flux in CT. The diagonal component of the influence matrix, known as self-sensitivity or observation impact, represents the impact of the observation on model value on each observation site.

**Fig 10:**
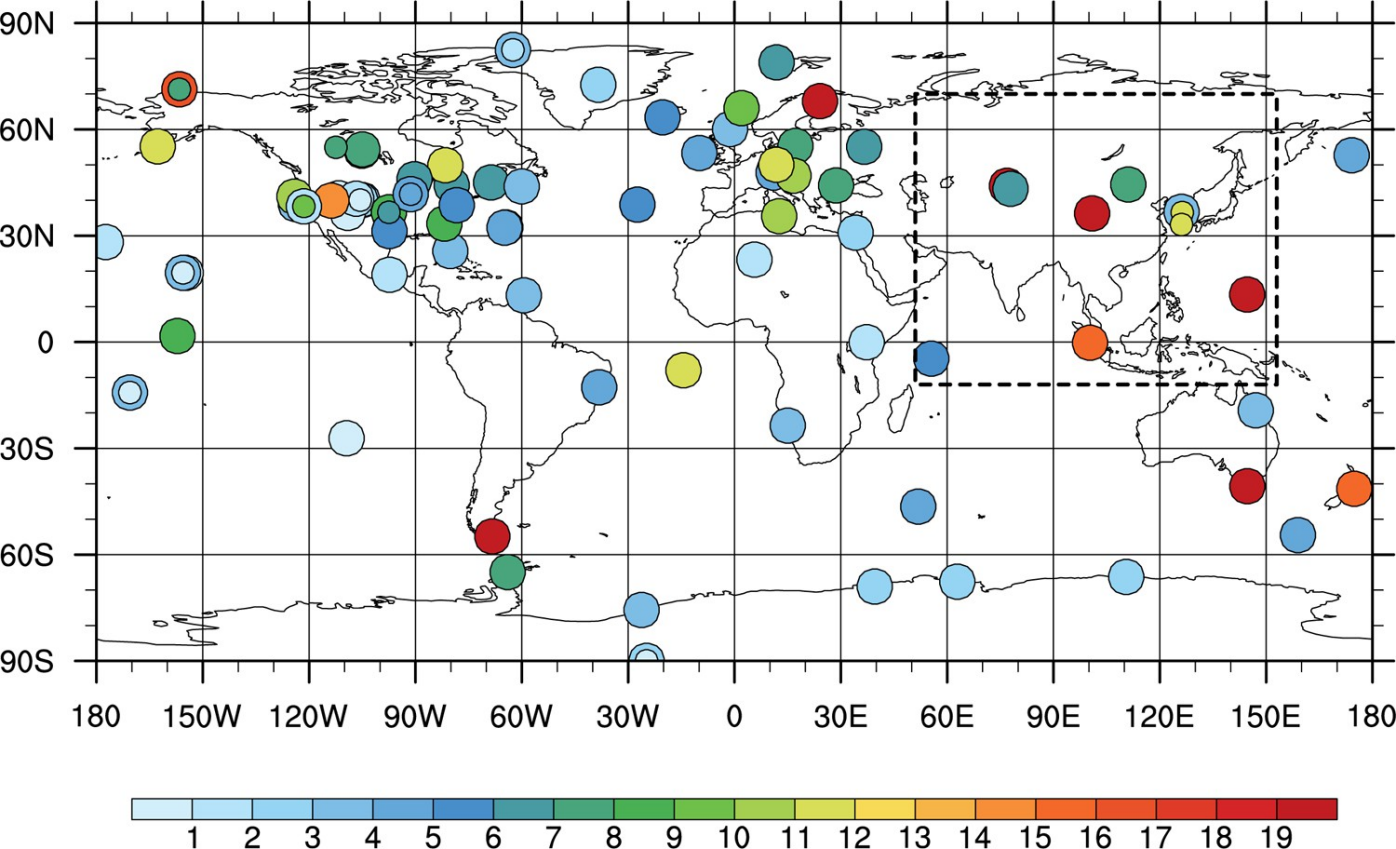
Average self-sensitivities at surface observation sites in the ObsPack and added sites in Korean Peninsula (AMY, GSN) for 2003–2011 period. The black box with dotted line is the nesting domain. The overlapping observation sites located at the same place or placed closely are distinguished by different sizes of circles.

Fig 10 shows the self-sensitivities of the surface CO_2_ observations assimilated in EXP1, averaged over the analysis period 2003–2011. AMY, GSN, and some overlapped sites are marked as circles with different size and color. The spatial density of observation sites is high in the North America and the Europe. The number of observation sites is relatively smaller in Asia, Australia, other continents, and oceans. Large self-sensitivities are found around observation-sparse regions. The self-sensitivities are fairly evenly distributed where observation sites are dense, although some sites in Alaska, Western America, and Northern Europe show large self-sensitivities. The global average self-sensitivity in EXP1 is 6.14%. The self-sensitivities of AMY and GSN are 11.71% and 11.38%, respectively, which are larger than the global average. Given that the self-sensitivity of TAP is 2.7%, the relatively large self-sensitivities in AMY and GSN imply that AMY and GSN observations play a more important role in producing the optimized surface CO_2_ flux. AMY and GSN observations also help estimate surface CO_2_ flux in Asia with low observation density.

## 4. Summary and conclusion

In this study, two CO_2_ observation datasets from AMY and GSN sites in the Korean peninsula are introduced in CT DA system and the effect of the observations on surface CO_2_ flux estimation in Asia is investigated for the 9-year period from 2003 to 2011. The annual average surface CO_2_ flux uptake on the East Asia is enhanced in EXP1 experiment in which AMY and GSN observations are assimilated. By assimilating observations from the AMY and GSN, ET regions including the Korean peninsula, Japan, and inland China show stronger CO_2_ absorption in summer, while weakened CO_2_ emission in spring and autumn. EB regions also show the similar pattern.

Independent surface and aircraft CO_2_ observations are used for the verification of the experimental results. Assimilating two additional observation datasets into CT reduced the root mean square error of modeled CO_2_ concentration with respect to independent CO_2_ observation concentration, and enhanced uncertainty reduction when optimizing surface CO_2_ flux in Asia region. The regions with small RMSEs are consistent with the regions with significant uncertainty reduction, which include the Korean Peninsula, southern inland China, eastern China, and Japan.

Self-sensitivities at AMY and GSN are relatively high, which indicates that the two observation sites in Korea (AMY, GSN) are considerably important in estimating surface CO_2_ flux in Asia. The use of CO_2_ observations in the Korean Peninsula is expected to greatly contribute not only to the estimation of surface CO_2_ flux in Asia at various scales, but also to the elaboration of the national emission inventory.
